# Ghosts from the past: a review of fossil Hepialoidea (Lepidoptera)

**DOI:** 10.7717/peerj.7982

**Published:** 2019-11-11

**Authors:** Thomas J. Simonsen, David L. Wagner, Maria Heikkilä

**Affiliations:** 1 Natural History Museum Aarhus, Aarhus, Denmark; 2 Department of Ecology and Evolutionary Biology, University of Connecticut, Storrs, CT, United States of America; 3 Finnish Museum of Natural History, University of Helsinki, Helsinki, Finland

**Keywords:** Calibration points, Evolutionary history, Fossil record, Morphology, *Oiophassus*, *Oxycanus*, *Prohepialus*, *Protohepialus*, *Sthenopis*

## Abstract

We critically re-examine nine of the ten fossil specimens currently assigned to Hepialidae. Three fossils with impressions of wing veins and scales placed in the fossil genus *Prohepialus* Piton, 1940, and two mummified larvae that show apomorphic characters, have features that support placement in Hepialidae. The other four fossils that we evaluate, *Prohepialus incertus* Piton, 1940; *Oiophassus nycterus* Zhang, 1989; *Protohepialus comstocki* Pierce, 1945; and a fossil scale, lack definitive hepialid characters. One of these, *Prohepialus incertus* Piton, 1940, appears to represent a symphytan (Hymenoptera), and is excluded from Lepidoptera. The fossilized wings placed in *Prohepialus* by Jarzembowski display numerous features that indicate a proximate phylogenetic relationship to extant members of the hepialid genus *Sthenopis* Packard and related genera.

## Introduction

Hepialoidea, commonly known as ghost moths, are the most species rich and ecologically diverse group within the homoneurous glossatan moths (Nielsen, Robinson & Wagner, 2000; Kristensen, 1998; Regier et al., 2015; Simonsen, 2018). The superfamily has a cosmopolitan distribution with the exception of Madagascar and Antarctica, and is most speciose in the Southern Hemisphere with major diversity centers in southern South America, southern Africa, and the Australian region (Kristensen, 1998; Grehan & Rawlins, 2016; Simonsen & Kristensen, 2017). Hepialoidea are often divided into two superfamilies in the suborder Exoporia (the other being the New Zealand endemic Mnesarchaeoidea). In this framework, Hepialoidea are classified in five families: Palaeosetidae, Prototheoridae, Neotheoridae, Anomosetidae, and Hepialidae (Kristensen, 1998; Nielsen, Robinson & Wagner, 2000; Nieukerken et al., 2011; Grehan & Rawlins, 2016). Here we follow the classification originally suggested by Scoble (1992) and upheld by Regier et al. (2015) and Simonsen & Kristensen (2017), which synonymise Neotheoridae, Anomosetidae, Prototheoridae, and Palaeosetidae with Hepialidae, and include Mnesarchaeidae as one of two families in Hepialoidea. Hepialoidea currently comprises more than 650 species in 70 genera (Nielsen, Robinson & Wagner, 2000; Simonsen, 2018).

A recent divergence time study estimated the hepialoid lineage to have originated in the mid-Jurassic, and Hepialidae in the mid-Cretaceous ca 95 million years ago (Wahlberg, Wheat & Peña, 2013). However, all known fossils assigned to Hepialoidea are much younger (Sohn et al., 2012). The oldest putative hepialoid fossil, *Prohepialus incertus* Piton, 1940, is from the mid-Paleocene (Selandian, 61.6–59.2 Ma) (Sohn et al., 2012) (although we assign that fossil to Hymenoptera, Symphyta, below). In addition to being relatively young, the fossil record of the superfamily is poor. Only ten fossil specimens (six records) have been assigned to Hepialoidea, all of them placed in the largest family of the superfamily, Hepialidae, some only tentatively (Sohn et al., 2012; Sohn, Labandeira & Davis, 2015). The relatively young and scarce fossil record is a general feature of Lepidoptera, often explained by the low probability of butterflies and moths to fossilize, related to their ecology, demographics, and weakly sclerotized bodies. Sohn et al. (2012) found 667 literature records for 4,568 fossil or subfossil lepidopterans. However, only 984 (21.5%) of these specimens have been assigned to an extant lepidopteran superfamily. The difficulty of identifying fossil Lepidoptera to superfamily or lower taxonomic levels is explained at least partly by the diagnostic characters often being hidden by scales or being located in body parts rarely visible in lepidopteran fossils (e.g., genitalia or skeletal sclerites).

The fossil record is plagued by misidentifications and taxonomic assignments, which can neither be verified nor refuted, making unfiltered use of the published literature or the Paleobiology Database (https://paleobiodb.org/#/) problematic. Recent reviews of fossils assigned to several different lepidopteran superfamilies have confirmed that much too often identifications are based on overall similarity to modern species, and not on apomorphies or unique character combinations (Doorenweerd et al., 2015; de Jong, 2017; Heikkilä et al., 2018; Heikkilä, Simonsen & Solis, 2018). Because fossils are increasingly used as calibration points in divergence time analyses—used to infer times of origins and date biogeographic events—we thought it would prove useful to review the veracity of determinations that have been applied to Hepialoidea. This study is part of an ongoing international collaboration to review the identifications of all known fossil Lepidoptera; here we re-examine the identifications of the hepialoid fossils listed in Sohn et al. (2012). We use the most recent information on the systematics and morphology of the superfamily to assess which of the current identifications are based on reliable evidence.

## Materials & Methods

### Specimen examination

Eight of the ten fossil specimens placed in Hepialoidea (Sohn et al., 2012) are compression or impression fossils of adult insects: a whole body fossil *Prohepialus incertus* Piton, 1940; six specimens with wings or wing fragments (*Oiophassus nycterus* Zhang, 1989; *Protohepialus comstocki* Pierce, 1945; a set of four fossils placed in the fossil genus *Prohepialus* sp. by Jarzembowski, 1980); and a wing or body scale attributed to Hepialidae in Evans (1931). Two are mummified larvae (Keble, 1947; Gill, 1955). No amber inclusions or trace fossils of Hepialoidea are known.

The ages of the fossils are given in Results and are adopted from the recent catalogue of fossil and subfossil Lepidoptera by Sohn et al. (2012).

Unfortunately, we were unable to obtain specimens on loan or visit the institutions to examine them directly (the ten fossils are deposited in six countries on four continents). Curators at those institutions that responded to our loan requests, consider the specimens too valuable to risk shipment. Our evaluations are based on the original descriptions, illustrations, and for *Prohepialus incertus* Piton, 1940, three of the four *Prohepialus* sp. fossils, *Protohepialus comstocki* Pierce, 1945, and two mummified larvae, on newly obtained, high-resolution photographs sent to us by the curators at the respective institutions. While this approach is much less optimal than examining the actual fossils, it still allowed us to evaluate nine of the ten fossils in the light of the most current knowledge on Hepialoidea and other relevant taxa, and share the images with colleagues at other institutions.

### Character observation

Most hepialids are many times larger than other homoneurans, but they are not larger than many heteroneurans, e.g., cossids, bombycoids, and some noctuoids. Characters defining adult Hepialidae include an evolutionary progressive regression of the haustellum (Simonsen & Kristensen, 2017); an elongate antennal intercalary sclerite; antennae that are often markedly short; on the wings, the posterior Rs fork is displaced caudad, and Rs3 reaches the margin, posterior to the apex; the wing scales usually have pronounced secondary ridges and large fenestrae; and metafurca lack an anterior process (except in the early diverging ‘neotheorid’ lineage) (Simonsen & Kristensen, 2017). Both male and female genitalia are highly modified in Hepialoidea compared to other Lepidoptera. Males possess a hinged juxta and trulleum sclerites, the tegumen (sclerotisation of tergum 9) is strongly reduced or absent—replaced by a sclerotisation of tergum 10 (pseudotegumen), the phallus is usually entirely membranous; and the female genitalia are of ‘exoporian’ type (but see (Kristensen & Nielsen, 1994) for modified configuration in *Osrhoes* Druce) (Nielsen & Kristensen, 1989; Kristensen, 1998).

Hepialidae larvae share numerous diagnostic features that allow their identification. Their large size alone will often distinguish them among all other homoneurans—the majority are over two cm, and most attain lengths greater than three cm in the last instar. With the exception of the head and pinacula, the body is mostly devoid of pigmentation and sclerotisation; in some, there are integumental microtrichia. The stemmata are often grouped into two vertical rows. An enlarged prothoracic shield reaches nearly to the prothoracic spiracle, and includes both SD and all three L setae; D1, XD1, XD2, L1, and L2 nearly form a line near the front edge of shield; SD1, SD2, and D2 are often grouped into a shallow, melanized depression. On A1–A8, the SD1 and SD2 setae sometimes share a common pinaculum, with SD2 about one-third length of SD1. Even though the larvae tend to be borers or subterranean, they are remarkably capable of both rapid and long-distance movements. As such, they have muscular prolegs; on the planta, the uniserial or biserial crochets are arranged in circles, with up to six or more rows in some larger taxa (e.g., *Sthenopis*). Other, mostly microscopic characters are given in Wagner (1987), but these generally would be difficult to view in fossils.

The above-mentioned diagnostic characters are not often visible in lepidopteran fossils because they are extremely small, hidden by scales, or internal structures. However, if a fossil forewing can be identified as a homoneuran, it is sometimes possible to see whether the Rs3 vein is displaced caudad—in which case the moth can be identified as a hepialid. The position of Rs3, and to a lesser extent Rs2, are the most important characters for identifying fossil hepialid wings. Wing scales of Hepialidae are not appreciably larger than what are found in similar-sized cossids, noctuoids, etc. (Simonsen & Kristensen, 2003), but the characteristic ultrastructure of the scales of Hepialoidea may be preserved as shown by Jarzembowski (1980). The much-abbreviated antennae can lend additional support to the identification of a moth fossil as a hepialid.

## Results

Below we discuss the character evidence for each of the nine fossils examined, from oldest to youngest.

**Table:** 

***Prohepialus incertus*** Piton, 1940 Fig. 1.

**Excavation locality and depository:** France: Cantal, Menat, Puy-de-Dôme (spongio-diatomite beds)/Selandian, Middle Paleocene. MNHN Holotype: MNHN.F.R07025 (old number: 426).

**Published illustrations:** Piton (1940): 217, pl. 17: 1 (black and white photograph).

**Condition:** Compression/impression fossil (adult: whole body in dorsolateral view). Length of body 33 mm, thorax at its widest 11 mm. According to Piton (1940), the wing venation is poorly preserved and only that of the right wings is partly visible. The length of the left forewing appears to be *ca* 30 mm [the apical region is missing]. A detailed description was provided by Piton (1940).

**Comments:** Piton found the visible part of the wing venation to resemble the venation of the extant genus *Hepialus* Fabricius, although we regard the details of the forewing to be too poorly preserved to be evaluated with certainty. Carpenter (1992) considered the assignment of this fossil to Hepialidae as suspect. Joël Minet at the MNHN and Conrad Labandeira (National Museum of Natural History, Smithsonian Institution) (both pers. comm., 2018) regard the impression fossil to be too ambiguous to allow a definitive taxonomic assignation.

The fossil displays many features that are incongruous for Lepidoptera. The body is too robust for a moth: the head is too broad from side-to-side; the near fusion of the head to the thorax, and especially broad joining of the thorax to the abdomen are at odds with those of Lepidoptera. The enormous abdomen, both in height and width, compounded by its abbreviated length are wrong for Lepidoptera. The abdominal proportions alone falsify the fossil as a hepialoid, which have abdomens >2–3 × longer than their width, and taper posteriorly. The antennae are as long as the head and thorax combined, which is longer than what is found in any current member of Hepialidae, including the early diverging lineages except for the males in some subordinate *Fraus* Walker species (e.g., *F. serrata* Nielsen & Kristensen, *F. distispina* Nielsen & Kristensen, *F. basidispina* Nielsen & Kristensen), but they all have a much less robust head and body. The very strong occipital? sulcus is puzzling and novel. Compound eyes are conspicuous components of the relief of primitive moth heads—they appear to lack appreciable relief in the *Prohepialus* impression. Because there is no evidence to support the identification of this specimen as a lepidopteran, we exclude it from the order.

David Grimaldi (pers. comm., 2018) suggested that the fossil is reminiscent of a horntail (Hymenoptera: Siricidae). Indeed, the venation in the hindwing more closely resembles that of a sawfly than a lepidopteran. Both the shape of the scape and antennal length are similar to those of a horntail. We concur with Grimaldi’s assessment, and suggest that the fossil be treated as a symphytan wasp.

**Figure 1:**
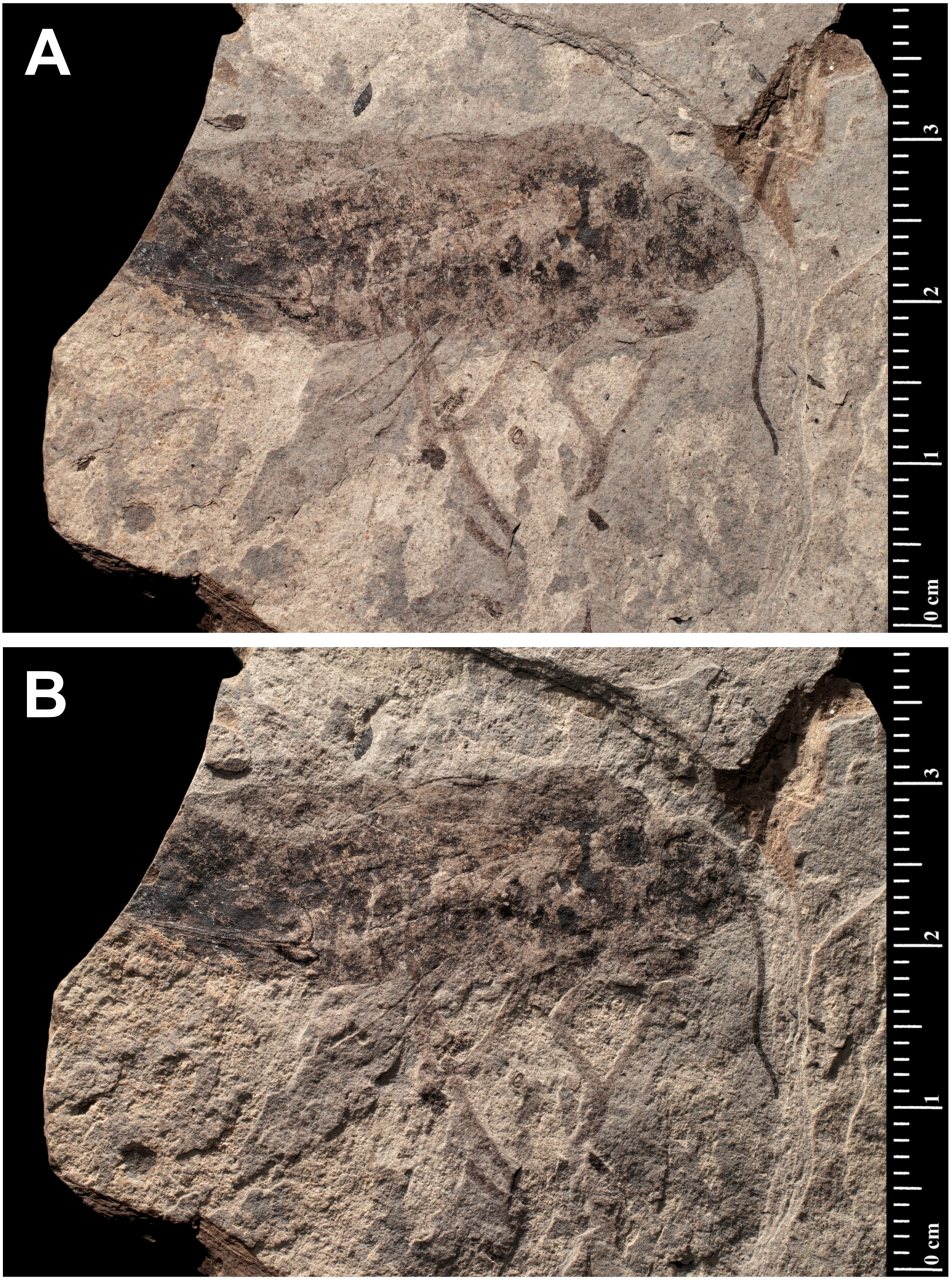
*Prohepialus incertus* Piton, 1940 (Holotype MNHN.F.R07025). (A) and (B) Specimen photographed under different lighting conditions. Photo credit: Gaëlle Doitteau (e-recolnat Project, MNHN).

**Table:** 

**Figure 2:**
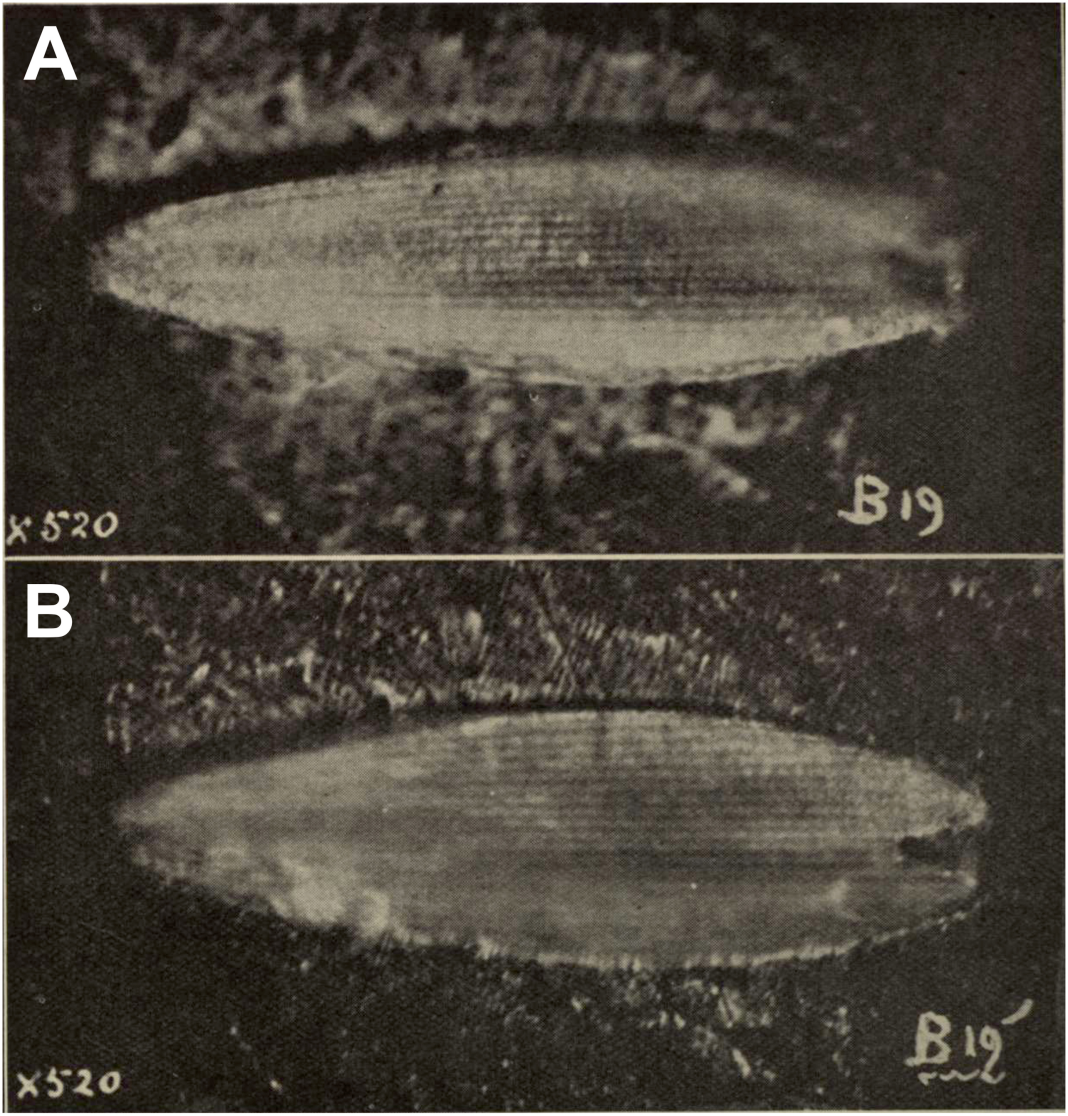
Fossil scale from Glen Afton coal. (A) and (B) Fossil scale with different portions in focus. Image not reproduced in original size. The length of the fossil scale given as 0.16 mm in Evans (1931). Reproduced with permission, the Royal Society of New Zealand.

**A scale attributed to Hepialidae in Evans (1931)**Fig. 2.

**Excavation locality and depository:** New Zealand: North Island, Waikato, near Huntly, Glen Afton mine (Waikato Coal Measures)/Priabonian, Late Eocene. The depository of this specimen is not known. It was not found in the Geological Collection of University of Auckland (N Hudson, pers. comm., 2018), National Paleontology Collection at GNS Science (M Terezow, pers. comm., 2018), or in the collections of the University of Otago (D Lee, pers. comm., 2018), Canterbury Museum (P Scofield, pers. comm., 2018), or Museum of New Zealand Te Papa Tongarewa (however, some of the material used in comparing the fossil to scales of extant hepialids, tineids, and other families, prepared by A. Castle and E.A. Plank (acknowledged in Evans, 1931), may be deposited there (P Sirvid, pers. comm., 2018).

**Published illustrations:** Evans (1931): 99, pl. 12 (black and white photographs).

**Condition:** Compression/impression fossil in coal. Length 0.16 mm.

**Comments:** A squamose wing scale, purportedly that of a hepialid, was figured by Evans (1931, figs B19, B19’). Other scales were present in the same deposit, but only a single example was imaged. R. J. Tillyard, a brilliant, well-trained entomologist and paleontologist, was primarily responsible for the interpretation offered by Evans. Tillyard felt the scale closely approached those of *Wiseana signata* (Walker), in shape, size (0.16 mm), and design, so much that Evans (1931) provided an image of a *W. signata* forewing scale for comparison in his publication.

The fossil scale lacks obvious synapomorphic features that would allow us to assign it to a lepidopteran family with certainty. It is not standard for hepialids: i.e., it has a deep apical invagination; evidently lacks obvious lacunae (as none were noted by either Evans or Tillyard); and lacks consistently spaced, well-differentiated primary and secondary ridging. Typical wing scales for the 30+ hepialid genera that we have examined (Wagner & Tindale, 1988; Simonsen, 2001; Simonsen, 2002; Simonsen, 2018; Kaaber, Kristensen & Simonsen, 2009; Simonsen & Kristensen, 2017; T Simonsen, D Wagner, pers. obs., 2001–2018) typically bear 3–7 apical teeth; have prominent windowing (more so than is evident in the Evans’s scales); and possess diagnostic, regular, well-differentiated primary and secondary ridges. The fossil scale figured by Evans (1931) lacks secondary ridging. Yet, secondary (larger) ridges are visible in the wing scale of the museum specimen of *W. signata* figured by Evans. We examined several hundred forewing scales of four *Wiseana* species, including those of *W. signata*, and did not find any with the deep apical cleft of the fossil scale (Evans, 1931, fig. B19’).

In sum, we consider the family assignment to Hepialidae uncertain. Other than the scale’s size and general shape, we see little structural evidence that the scale belongs to a hepialid, and the preponderance of features that would allow its assignment to Hepialoidea are either lacking or not discernible from the images in Evans’s (1931) and Tillyard’s assessment. We, like Tillyard, feel it represents a lepidopteran, and possibly a hepialid, but there were other lineages present in the Late Eocene that might have been the source of the scale: it could equally well belong to a Neopseustidae or represent a fringe scale from a lower Ditrysia (e.g., Simonsen, 2001; Kristensen & Simonsen, 2003).

**Figure 3:**
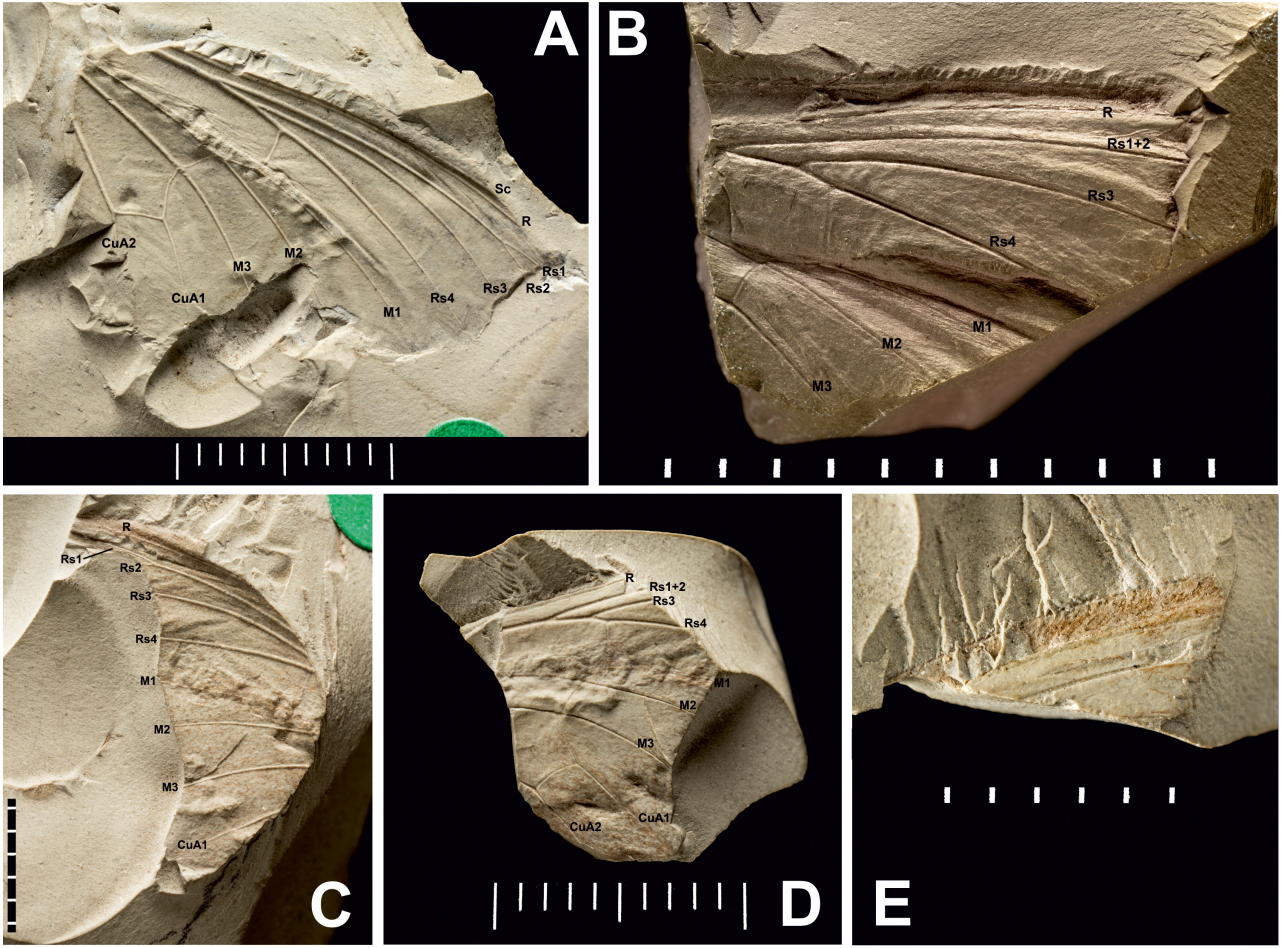
Three *Prohepialus* sp. fossils identified by Jarzembowski (1980). (A) Incomplete wing, In17464. (B) Incomplete wing, mainpart In64538 (i) (the counterpart (not photographed) is on a fragment of In 64528). (C) Incomplete wing, mainpart In64528 (i). (D) Incomplete wing, In64528 (ii) (counterpart fragment of In64528 (i)). (E) In64528 (iii) (counterpart fragment of In64528 (i)). Scale in mm. Photo credit: Harry Taylor, Natural History Museum, London.

**Table:** 

**Figure 4:**
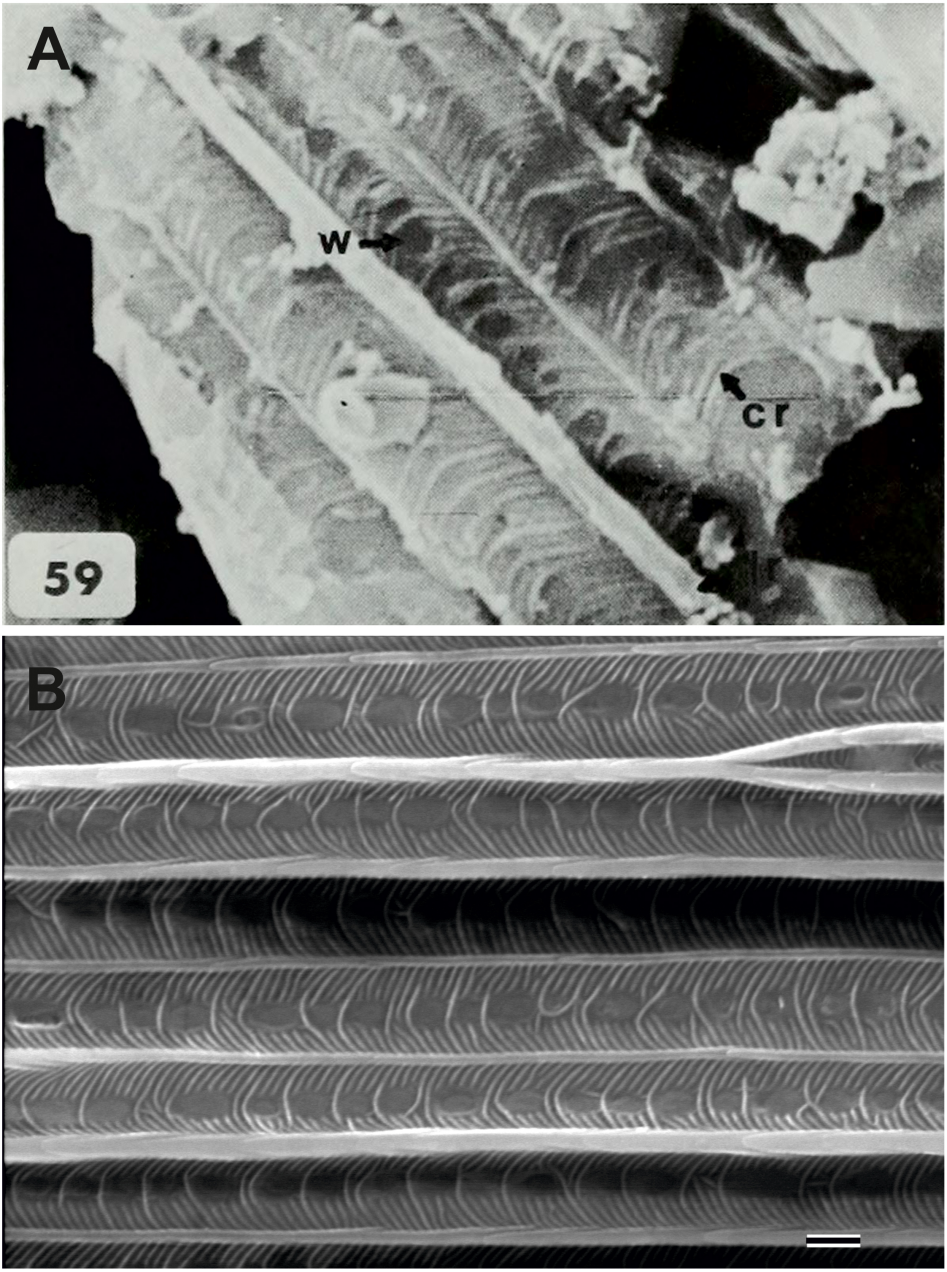
Scanning electron microscope images of *Prohepialus* sp. identified by Jarzembowski (1980) and *Sthenopis argenteomaculatus* (Harris). (A) SEM image of *Prohepialus* sp. identified by Jarzembowski (1980) with details of scale ultrastructure. w = window; cr = crossrib; lr = longitudinal ridge (in lower right corner). Reproduced with permission, ©The Trustees of the Natural History Museum, London. (B) *Sthenopis argenteomaculatus* scale ultrastructure, pale olive-brown forewing scale, abwing surface (surface of the scale facing away from the wing membrane) of dorsal side scale, ZMUC Hep st80. Scale 1  μm. Image credit: Thomas Simonsen.

**Wing fragments assigned to the fossil genus** ***Prohepialus*** **Piton, 1940 by Jarzembowski (1980)** Figs. 3 and 4.

**Excavation locality and depository:** United Kingdom: England, Isle of Wight, Bembridge Marls (Bouldnor Fm.)/Late Priabonian, Late Eocene. NHMUK, the material consists of four incomplete wings (a total of six fragments with impressions of wings on one or two sides): In.17464; In.64528 (i) and fragments of counterpart In.64528 (ii–iii); In.64538 (i) and counterpart on reverse side of In.64528 (ii); In.64539 (H). We were able to examine newly-taken, high-resolution photographs of three of the four wings (In.17464, In.64538 (i), and In.64528 (i–iii)).

**Published illustrations:** Robinson (1977): 109, fig. 5 (In.17464) and fig. 6 (In. 64528) (drawings); Jarzembowski (1980): 265, figs 38 (In.17464), 47 (In.64528), 59 (In.64538) (photograph, drawing and scanning electron micrograph of scale, respectively). Both Robinson (1977) and Jarzembowski (1980) are available in the Biodiversity Heritage Library (https://www.biodiversitylibrary.org/).

**Condition:** Compression/impression fossils of partial forewings or partial hindwings and wing scales. Jarzembowski (1980) estimated the complete wing lengths to be 20–45 mm, and suggested the shorter value would apply were the wings shown to represent hindwings. Width of discal cell across ca. 4.4–9.8 mm, In.64538–9. It is not known from how many individuals the wings are, but Jarzembowski (1980) suggested that In.64538 and In.64528 could be the fore- and hindwings of the same individual as they come from the same piece of rock.

**Comments:** The fossils were first mentioned in Jarzembowski (1976) and briefly discussed by Robinson (1977) who saw a resemblance in the venation with the genus *Callipielus* Butler and other extant hepialid genera. The fossils were subsequently described in more detail by Jarzembowski (1980) who drew attention to the M-vein, which is forked inside the cell, the oblique m-cu cross vein, and the overall large size as proof of a placement within Hepialidae. While we agree that these characters are suggestive of their placement as Hepialidae, none are conclusive: an M-vein, which is forked inside the cell, occurs sporadically in non-ditrysian Heteroneura and lower Ditrysia (e.g., Davis, 1998; Davis & Robinson, 1998); the shape of the m-cu varies considerably both in the Hepialidae and across lower Lepidoptera (e.g., Kristensen, 1998; Davis, 1998; Davis & Robinson, 1998; Simonsen, 2018); while most Hepialidae are indeed large, other groups (which display the venation characters listed above) such as Cossidae and Andesianidae are of similar size (Edwards et al., 1998; Davis & Gentili, 2003).

Nevertheless, we concur that specimens In.17464, In.64538, and In.64528 are likely wings of a hepialid. Although the wings are incomplete, Jarzembowski’s (1980) fig. 47 shows the overall venation and clearly indicates that Rs3 (R4 in his figure) would reach the wing margin caudal to the wing apex if the wing were complete. In fact, our interpretation is that even Rs2 (R3 in the figure) would reach the margin either caudal to the apex or at the apex. According to Kristensen (1998) and Simonsen & Kristensen (2017), an Rs3 that reaches the margin caudal to the wing apex is an apomorphy for Hepialidae (Hepialoidea in Kristensen (1998), an Rs2 that reaches the margin at or caudal to the apex is an apomorphy for a clade comprising the ‘palaeosetid’ genera, the ‘primitive hepialids’ (*Fraus*, *Gazoryctra* Hübner, *Afrotheora* Nielsen & Scoble, and *Antihepialus* Janse), and Hepialidae *s. str.*, but excluding the South American ‘neotheorid’ genera (*Neotheora* Kristensen and *Paratheora* Kristensen & Simonsen), the Australian genus *Anomoses* Turner, and the South African genus *Prototheora* Meyrick.

Jarzembowski (1980, fig. 59) further illustrated a scanning electron micrograph of a wing scale (most likely showing the abwing surface of a scale from the dorsal wing surface) from the fossil, which he argued supported this placement, as the scale has large windows and a pronounced dimorphism in the longitudinal ridges. We fully agree. Although ridge dimorphism is widespread in the non-ditrysians, pronounced secondary ridges as seen in the figure are only found in the hepialid clade also characterized by the backwards placement of both Rs2 and Rs3 (Simonsen, 2001; Simonsen, 2018). The shape of the windows and cross ribs further point towards a close relationship with the ‘primitive hepialids’ and Hepialidae *s. str.*, as the ‘palaeosetid’ genera have specialized windows often with thick frames, and a single, well-defined cross rib between each window or group of windows (Simonsen & Kristensen, 2017).

We compared the SEM image of Jarzembowski’s *Prohepialus* (Fig. 4A) to images of the abwing surfaces of dorsal scales from extant hepialoids in the genera *Abantiades* Herrich-Schäffer, *Aenetus* Herrich-Schäffer, *Afrotheora, Andeabatis* Nielsen & Robinson, *Anomoses, Antihepialus, Archaeoaenetus* Simonsen, *Bipectilus* Chu & Wang, *Callipielus* Butler, *Dalaca* Walker, *Druceilla* Viette, *Elhamma* Walker, *Fraus, Gazoryctra, Genustes* Issiki & Stringer, *Gorgopis* Hübner, *Hepialus, Korscheltellus* Börner, *Mnesarchaea* Meyrick, *Ogygioses* Issiki & Stringer, *Oncopera* Walker, *Osrhoes, Oxycanus* Walker, *Neotheora, Palaeoses* Turner, *Parapielus* Viette, *Paratheora, Pharmacis* Hübner, *Prototheora* Meyrick, *Phymatopus* Wallengren, *Sthenopis, Trichophassus* Le Cerf, *Zelotypia* Scott (Kristensen & Nielsen, 1994; Simonsen, 2001; Simonsen, 2002; Simonsen & Kristensen, 2017; T Simonsen, pers. obs., 2001–2018) and found it to best approximate the scales of *Sthenopis argenteomaculatus* (Harris) (Fig. 4B). Forewing scales of a few taxa also examined, e.g., those of *Fraus minima* Nielsen & Kristensen, were also similar, but did not match as closely. The wing venation differs markedly from the latter, as Rs3 and Rs4 are branched with a common stem in *Fraus* (Nielsen & Kristensen, 1989), but originate independently on the cell in the fossil.

The forewing venation of the following taxa were compared to that of the fossils: *Hepialus humuli* (Linnaeus), *S. argenteomaculatus*, *S. pretiosus* Herrich-Schäffer, *S. purpurascens*, *S. thule* (Strecker), “*Sthenopis” ?bouvieri* (Oberthur), *“Sthenopis” regius* (Staudinger), and *Zenophassus schamyl* (Christoph). The near parallel veins of Rs1–Rs2 and Rs3 seems noteworthy (J Grehan in litt., 2019). The closest match with regard to subparallel Rs1–Rs2 and Rs3 veins was seen in just two taxa: *S. argenteomaculatus* and *S. pretiosus* with the second being exceeding close, in large measure because the Rs1–Rs2 fork is displaced distad in that taxon (see below). And by contrast, Rs3 is distinctly bent downward away from Rs1–Rs2 in *S. bouvieri* and *S. thule*, and *Zenophassus*. Rs3 also bends away from Rs1–Rs2, but more gradually, in *S. regius* and *H. humuli*.

Jarzembowski assigned the specimens to the fossil genus *Prohepialus* Piton, 1940 but as explained above, the weight of the evidence suggests that Piton’s genus is hymenopteran, and we recommend removing it from the Lepidoptera. Therefore, Jarzembowski’s specimens, which we agree show hepialid characters, cannot remain in *Prohepialus*. We are, however, of the opinion that the fossils are too incomplete to allow us to place them with confidence in an existing hepialid genus. The overall wing size, shape, relief of veins, relative thicknesses of veins and cross veins (to one another), and general venation closely agree with moths in the *Sthenopis* sensu lato group. The wing venation does resemble the genus *Sthenopis* although the short Rs1–Rs2 fork differ from *Sthenopis*, which have a longer Rs1–Rs2 fork (D Wagner, T Simonsen, pers. obs., 2018–2019)—the only exception being *S. pretiosus*, which have an even shorter Rs1–Rs2 fork than the fossils (D Wagner, pers. obs., 2019). Furthermore, *Sthenopis* (*sensu* Nielsen, Robinson & Wagner, 2000) may not be monophyletic (J Grehan pers. comm., 2019), and we therefore suggest that Jarzembowski’s set of fossils be listed near that genus as ‘*Sthenopis* cf. sp.’

**Figure 5:**
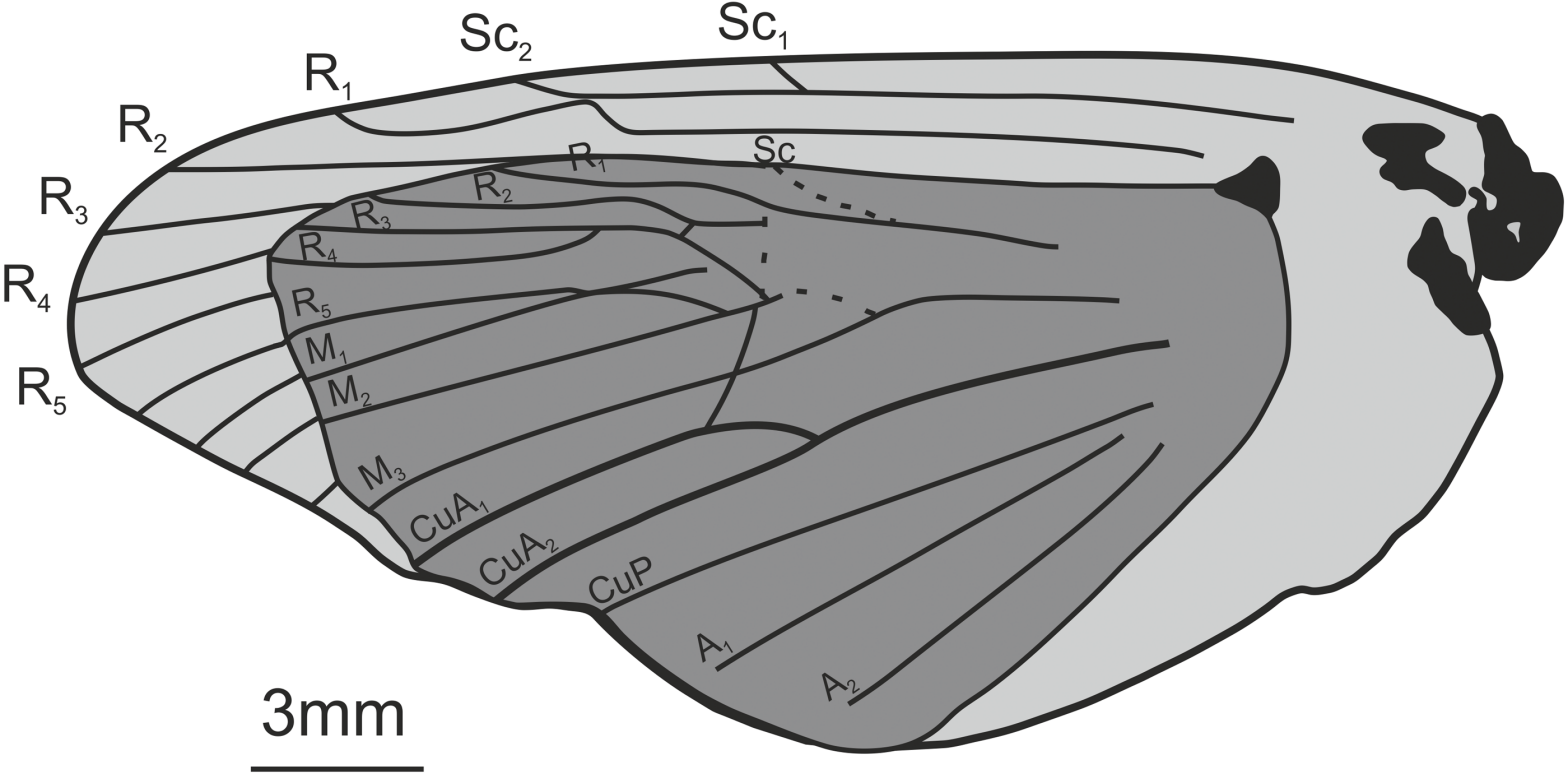
*Oiophassus nycterus* Zhang, 1989. Holotype s82702. Redrawn after Zhang (1989).

**Figure 6:**
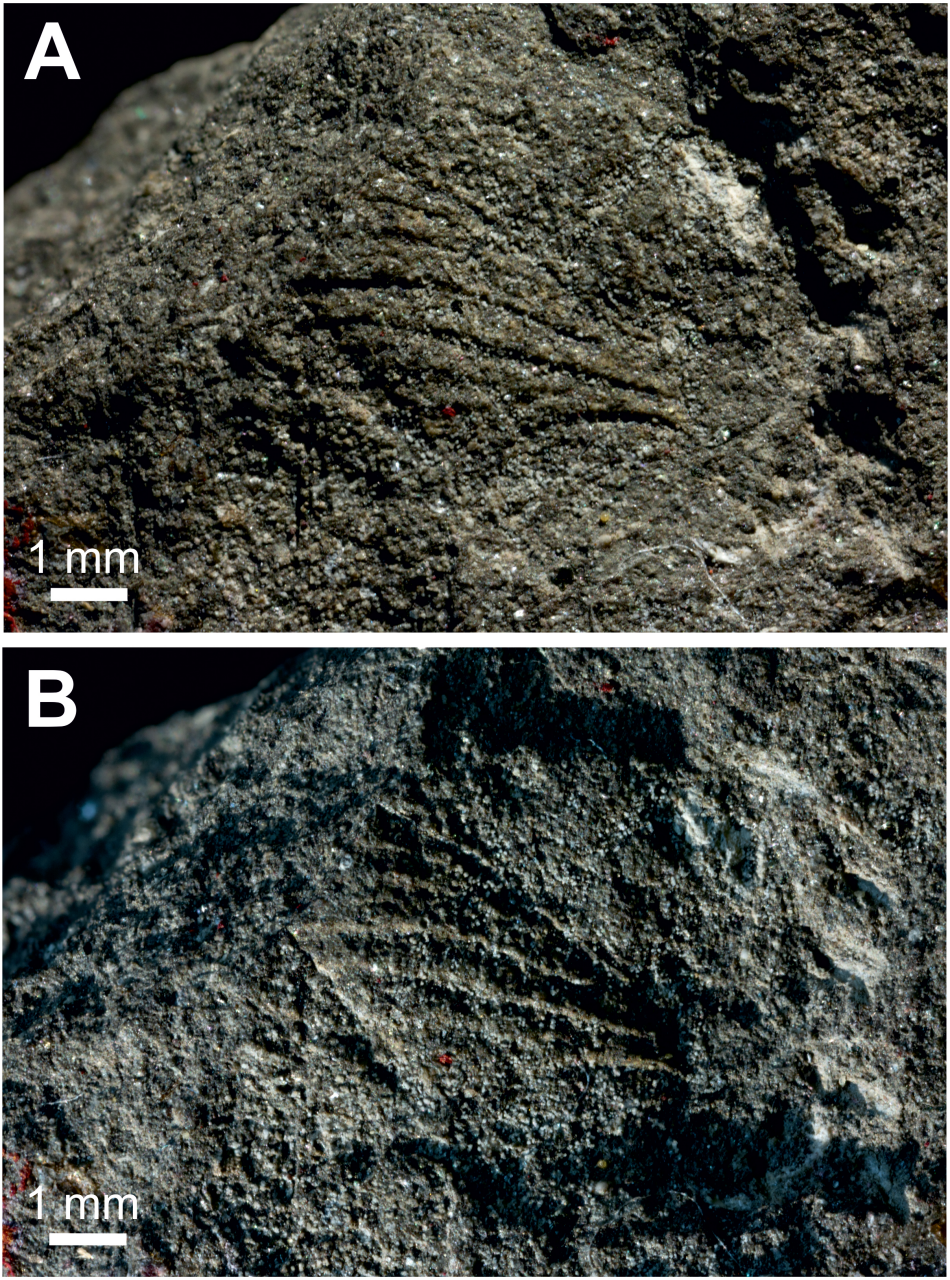
*Protohepialus comstocki*Pierce, 1945. Catalog number LACMIP 85.2, type number LACMIP Type 3072. (A) and (B) Specimen photographed under different lighting conditions. Photo credit: Natural History Museum of Los Angeles County.

**Table:** 

***Oiophassus nycterus*** Zhang, 1989 Fig. 5.

**Excavation locality and depository:** China: Shandong Prov., Lingu, Shanwang (Shanwang Fm.)/Langhian, Middle Miocene. According to Sohn et al. (2012) the specimen (holotype: s82702) is deposited in the SFML. We have not been able to confirm this information.

**Published illustrations:** Zhang (1989): 94, fig. 75 (drawing), pl. 20: 4 (black and white photograph).

**Condition:** Compression/impression fossil of superimposed fore- and hindwing. Forewing length ca 2.4 cm. Hindwing length 1.7 cm.

**Comments:** We consider it highly unlikely that this fossil belongs to Hepialidae, and even find a placement within the Lepidoptera doubtful. While the wings superficially resemble those of a homoneuran moth, several details speak against such an assignment. First, the Rs3 (R4 in the figure) is reaching the wing margin before the apex. Second, the subcosta (Sc) and the radius (R1 in the figure) merge and separate again shortly before the wing margin. While *Agathiphaga* Dumbleton generally have a distal cross vein between these veins (Schachat & Gibbs, 2016)—and it is not inconceivable that they could merge in some specimens—this condition is not known from other homoneuran moths (including Hepialidae). Finally, the wing has a distinct anal lobe that appears to be without veins in the basal half, which is not a condition known in non-ditrysian Lepidoptera. The first character is incompatible with placement in Hepialidae, while the combination of the latter two features speaks against placement within Lepidoptera. We refrain from suggesting an alternative order for the fossil, but note that the shape of the wings and the venation are reminiscent of many Hemiptera.

**Table:** 

***Protohepialus comstocki*** Pierce, 1945 Fig. 6.

**Excavation locality and depository:** USA: California, Los Angeles Co., SE Puente (Puente Fm.)/Late Miocene. NHLA, catalog number LACMIP 85.2, and the type number LACMIP Type 3072.

**Published illustrations:** Pierce (1945): 5, pl. 3 and 4 (black and white photograph and drawing).

**Condition:** Compression/impression fossil. Partial wing occupying a space about 5 ×5 mm.

**Comments:** In Skalski (1990) this fossil is called *Protohepialis* [sic] *incertus* Pierce, 1945. According to the original description the fossil is a “faint impression of a portion of a wing” (Pierce, 1945, p. 5).

We are dubious that this impression represents a lepidopteran wing. The relief of the veins is too great to belong to any lepidopteran with which we are familiar: the diameters of the veins are too broad to belong to a moth of this size. The indication in the fossil that is interpreted to be the SC vein is not clearly a vein in that it lacks relief, it appears to cross the radial vein (rather than anastomose with it), and its angle where it crosses the R vein appears too steep to be a subcostal juncture. The strongly arching putative Cu2 vein seems exceptional or anomalous and worthy of additional evaluation. Finally, the wing width to length ratio is questionable: the majority of primitive Lepidoptera have wings that are appreciably narrower than those of this impression fossil, in which the wing is nearly as wide as it is long. Given the relief of the veins (relative to the “wing” size), and other misgivings, we are not convinced this is an insect wing, and would not rule out that the fossil belongs to a plant or represents a swimming structure. Regardless, it is not assignable to Hepialidae.

**Table:** 

**Figure 7:**
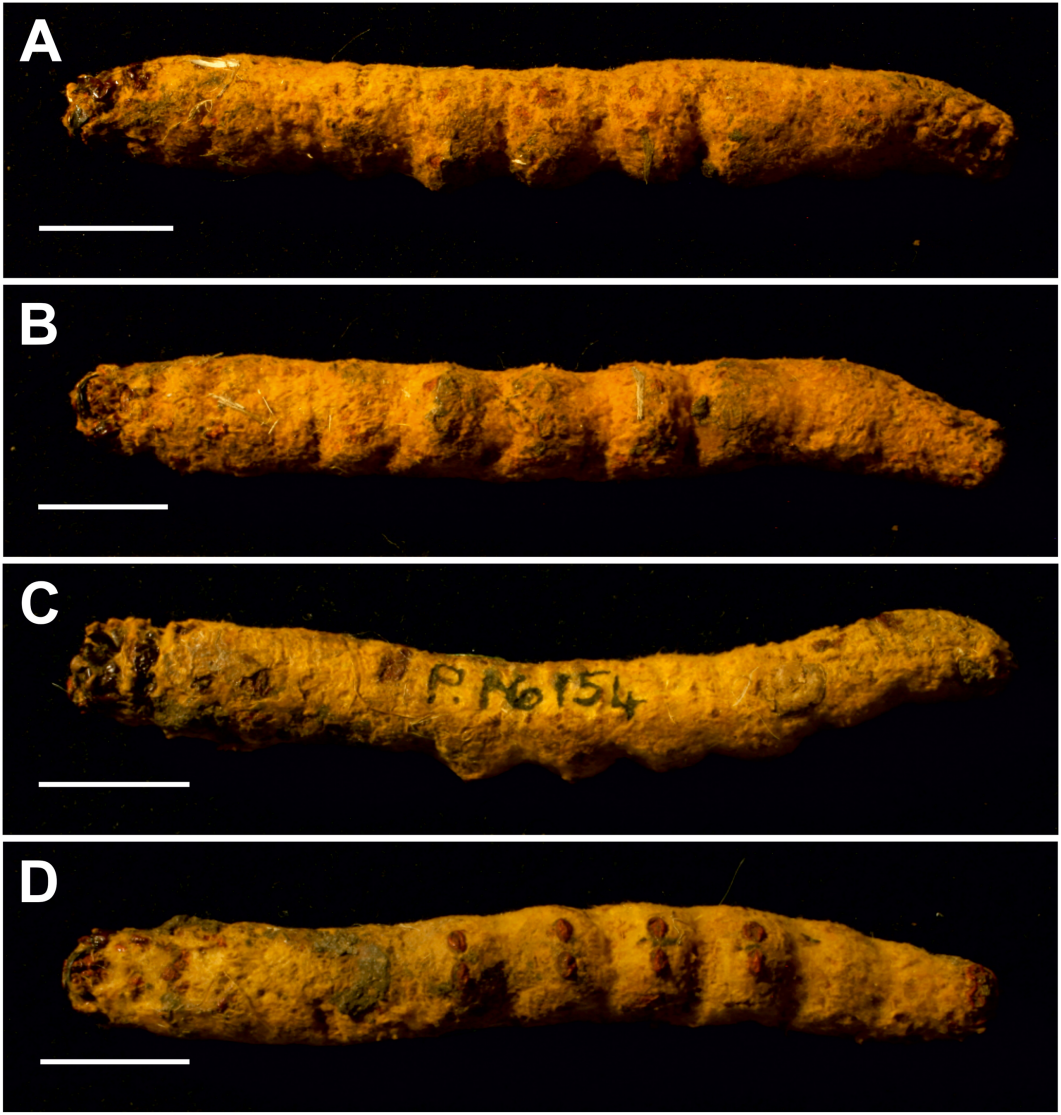
Mummified larvae from Pejark Marsh, Australia (P16153 and P16154). (A) Specimen P16153: lateral view. (B) Specimen P16153: ventral view. (C) Specimen P16154: lateral view. (D) Specimen P16154: ventral view. Scale bar one cm. Photo credit: Rolf Schmidt, Museum Victoria.

**Figure 8:**
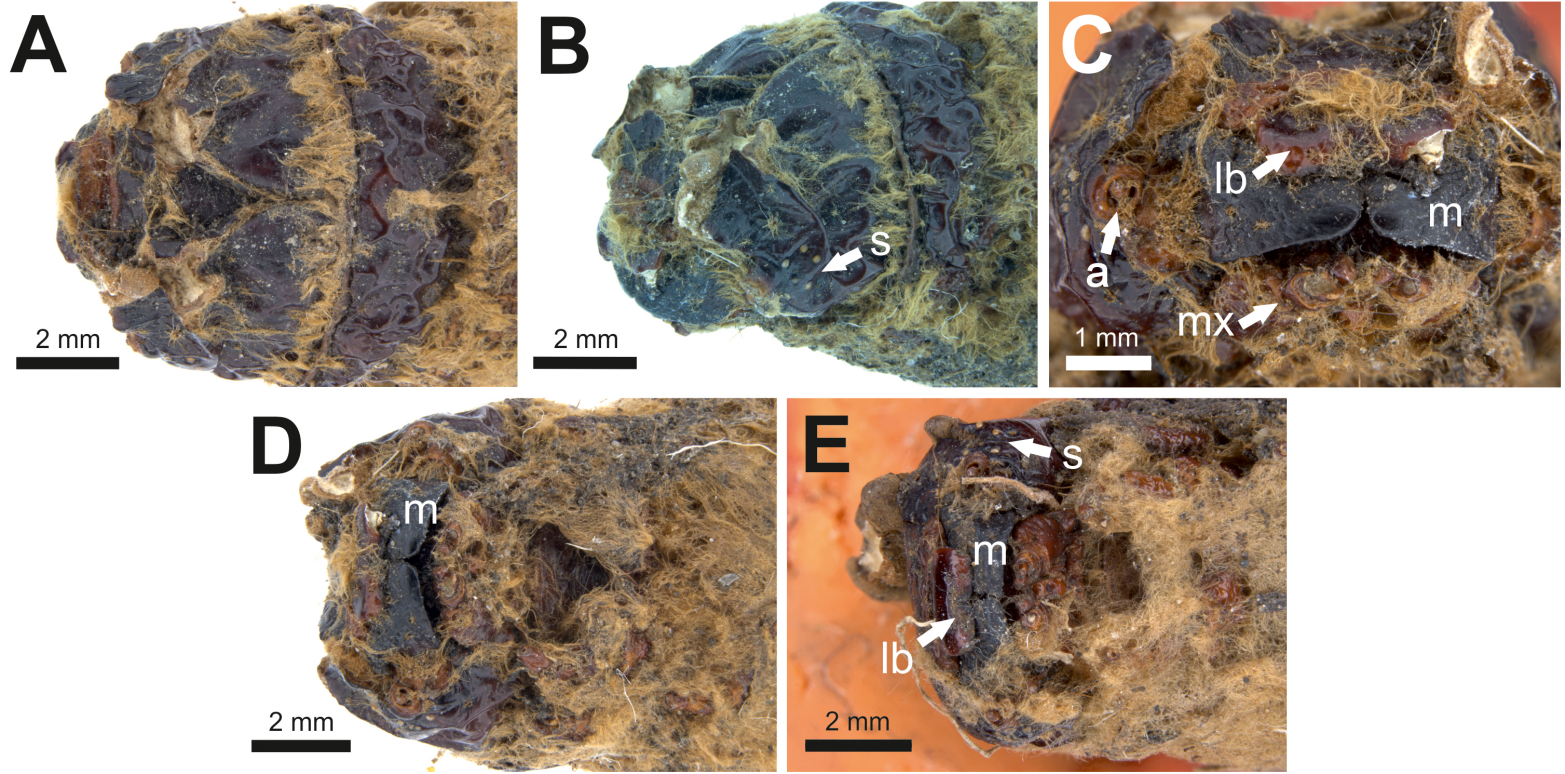
Head and mouthparts of two mummified larvae (P16153 and P16154). (A) Specimen P16153: dorsal view of head. (B) Specimen P16153: lateral view of head. (C) Specimen P16153: close-up of mouthparts. (D) Specimen P16153: ventral view of head. (E) Specimen P16154: ventral view of head. a = antenna; lb = labrum; m = mandible; mx = maxilla; s = stemmata. Photo credit: Rolf Schmidt, Museum Victoria.

**Mummified larvae*cf Oxycanus antipoda*** **(Herrich-Schäffer)** **mentioned in Keble (1947)and illustrated in Gill (1955)** Figs. 7 and 8.

**Excavation locality and depository:** Australia: Victoria, Pejark Marsh (unconsolidated sediments)/Late Holocene. MV, 2 specimens: P16153; P16154.

**Published illustrations:** Gill (1955): 88, pl. III (black and white photograph). Biodiversity Heritage Library: https://biodiversitylibrary.org/page/40868443.

**Condition:** Two mummified larvae, whole body. No other details given in Keble (1947). The length of specimen P16153 is ca seven cm, and of specimen P16154 ca 6.5 cm.

**Comments:** The fossils were identified by entomologist A. N. Burns (in Keble, 1947) as approximating *Oxycanus fuscomaculatus* Walker, junior synonym of *Oxycanus antipoda* (Herrich-Schäffer).

The body proportions and transverse abdominal creasing are both consistent with Hepialidae (Wagner, 1987). The stemmata are arranged in two vertical rows. The hypertrophied, muscular planta is diagnostic for hepialoids, and functionally related to their mobile lifestyle, allowing them to move rapidly, both forward and back, along their silken tunnels to feed, rest, and avoid enemies. Segments 7–10 are comparatively elongate in the mummified larvae (see Fig. 7): collectively longer than would be found in soil-dwelling noctuids or other subterranean macrolepidopteran larvae with which we are familiar. Both Gill and Burns offered an approximate species identification. Evidently, they felt certain about the genus and family assignment, given that each suggested a (different) species-level name.

## Discussion

Despite representing an ancient lineage in Lepidoptera—potentially dating back to the mid-Cretaceous (Wahlberg, Wheat & Peña, 2013)—Hepialidae are extremely rare in the fossil record, and only ten putative fossil specimens were associated with the family by Sohn et al. (2012). Our results show that the superfamily’s fossil record is even poorer than previously understood: only five of the nine fossils that we examined can be assigned to Hepialoidea with reasonable certainty. The set of fossils assigned to *Prohepialus* by Jarzembowski (1980), which we consider to be allied to *Sthenopis,* is the oldest, dating to the late Priabonian (ca 35 Ma). Although this is much younger than the age inferred for the origin of the family based on molecular dating methods (Wahlberg, Wheat & Peña, 2013), its overall similarity to modern-day *Sthenopis* and *Zenophassus* Tindale*,* does provide insight on the nature of phenotypic evolution within Hepialidae. For the purpose of calibrating molecular clock analyses, the fossil provides a potential minimum age for the *Sthenopis* group (Wagner & Rosovsky, 1991) but in the absence of other visible characters diagnostic of this group of moths, the assignment must be considered tentative and its use as a calibration point is not advised. But more importantly, the fossil provides new insight for understanding the historical biogeography and diversity of Hepialidae. *Sthenopis* as currently defined, includes four species restricted to North America, *Zenophassus* occurs in the Caucasus, and there are several *Sthenopis*-like species in China (Nielsen, Robinson & Wagner, 2000), but see comment above. However, no *Sthenopis-* like species currently occurs in the United Kingdom or elsewhere in the Western Palearctic.

The two fossil larvae are very recent (dated to Late Holocene) and appear to fit comfortably into *Oxycanus*—indeed two different entomologists assigned the fossils to extant species of the genus. But we caution that we have not yet had a chance to physically examine the specimens and compare these to larvae of extant peatland-inhabiting *Oxycanus* and other members of the genus.

The sparse fossil record of Hepialoidea is typical for lepidopterans, i.e., frustratingly depauperate (e.g., Sohn et al., 2012; Doorenweerd et al., 2015; De Jong, 2017; Heikkilä et al., 2018; Heikkilä, Simonsen & Solis, 2018). In addition to the general conditions affecting fossilization of Lepidoptera as discussed by these authors, the numerical scarcity of hepialoids (as adults) and high body-fat content (by increasing their buoyancy) may reduce the likelihood that they will be represented in the fossil record.

## Conclusions

The study contributes to efforts to obtain a more accurate and reliable picture of the evolutionary history and historical biogeography of Lepidoptera. Of the nine fossils specimens we re-examined, only three wing fragments from the Bembridge Marls, Isle of Wight, England, (Late Eocene) and two mummified fossil larvae from Pejark Marsh, Victoria, Australia (Late Holocene) display synapomorphies or character combinations diagnostic of Hepialidae. The other four putative hepialoid fossils lack definitive character evidence and cannot be placed in the superfamily with certainty. One of them (and the type species for the genus *Prohepialus*!) appears to represent a symphytan (Hymenoptera). Our results highlight the need to re-examine the identifications of fossil Lepidoptera that have not yet been reassessed in the light of recent knowledge of lepidopteran morphology and systematics, and underscore the importance of basing taxonomic assignments of fossils on more rigorous assessments of anatomical evidence.

##  Supplemental Information

10.7717/peerj.7982/supp-1 Supplemental Information 1 Depository and accession numbers of fossil specimens examined

## Institutional abbreviations

MNHN: Institute of Paleontology, National Museum of Natural History in Paris (= Institut de Paleontologie, Muséum National d’Histoire Naturelle de Paris), Paris, France
MV: Museum Victoria, Melbourne, Australia
NHLA: Natural History Museum of Los Angeles County (= Los Angeles County Museum), Los Angeles, California, U.S.A.
NHMUK: Department of Paleontology, Natural History Museum, London, United Kingdom
SFML: Shanwang Fossil Museum, Lingu Province, Shandong, China

## Additional Information and Declarations

### Data Availability

The following information was supplied regarding data availability: *Prohepialus incertus* Piton, 1940, is deposited at the Institute of Paleontology, National Museum of Natural History in Paris (MNHN.F.R07025); wing fragments assigned to the fossil genus *Prohepialus* Piton, 1940 by Jarzembowski (1980) are deposited at the Department of Paleontology, Natural History Museum, London, United Kingdom (In.17464; In.64528 (i) and fragments of counterpart In.64528 (ii–iii); In.64538 (i) and counterpart on reverse side of In.64528 (ii); In.64539 (H)); *Protohepialus comstocki* Pierce, 1945, is deposited at the Natural History Museum of Los Angeles County, USA (catalog number: LACMIP 85.2; type number: LACMIP Type 3072); Mummified larvae cf *Oxycanus antipoda* (Herrich-Schäffer) mentioned in Keble (1947) and illustrated in Gill (1955) are deposited at the Museum Victoria, Melbourne, Australia (P16153; P16154). Depository information for the fossil scale attributed to Hepialidae in Evans (1931) is not known, though some of the material used in comparing the fossil to scales of extant hepialids, tineids, and other families, prepared by A. Castle and E.A. Plank (acknowledged in Evans (1931), may be deposited at the Museum of New Zealand Te Papa Tongarewa. We have not been able to confirm depository information for *Oiophassus nycterus* Zhang (1989). According to Sohn et al. (2012) the specimen (holotype: s82702) is deposited in the Shanwang Fossil Museum, Lingu Province, Shandong, China. Depositories and accession numbers of fossil specimens examined are provided in File S1.
